# Sickness presenteeism in Spanish-born and immigrant workers in Spain

**DOI:** 10.1186/1471-2458-10-791

**Published:** 2010-12-29

**Authors:** Andrés A Agudelo-Suárez, Fernando G Benavides, Emily Felt, Elena Ronda-Pérez, Carmen Vives-Cases, Ana M García

**Affiliations:** 1 Centre for Research in Occupational Health, Universitat Pompeu Fabra, Barcelona, Spain; 2 Preventive Medicine and Public Health Area, University of Alicante, Alicante, Spain; 3 Faculty of Dentistry, University of Antioquia, Medellin, Colombia; 4 CIBER Epidemiology and Public Health, Spain; 5 Department of Preventive Medicine and Public Health, University of Valencia, Valencia, Spain; 6 Trade Union Institute for Work, Environment and Health (ISTAS), Spain

## Abstract

****Background**:**

Previous studies have shown that immigrant workers face relatively worse working and employment conditions, as well as lower rates of sickness absence than native-born workers. This study aims to assess rates of sickness presenteeism in a sample of Spanish-born and foreign-born workers according to different characteristics.

**Methods:**

A cross-sectional survey was conducted amongst a convenience sample of workers (Spanish-born and foreign-born), living in four Spanish cities: Barcelona, Huelva, Madrid and Valencia (2008-2009). Sickness presenteeism information was collected through two items in the questionnaire ("Have you had health problems in the last year?" and "Have you ever had to miss work for any health problem?") and was defined as worker who had a health problem (answered yes, first item) and had not missed work (answered no, second item). For the analysis, the sample of 2,059 workers (1,617 foreign-born) who answered yes to health problems was included. After descriptives, logistic regressions were used to establish the association between origin country and sickness presenteeism (adjusted odds ratios aOR; 95% confidence interval 95%CI). Analyses were stratified per time spent in Spain among foreign-born workers.

**Results:**

All of the results refer to the comparison between foreign-born and Spanish-born workers as a whole, and in some categories relating to personal and occupational conditions. Foreign-born workers were more likely to report sickness presenteeism compared with their Spanish-born counterparts, especially those living in Spain for under 2 years [Prevalence: 42% in Spanish-born and 56.3% in Foreign-born; aOR 1.77 95%CI 1.24-2.53]. In case of foreign-born workers (with time in Spain < 2 years), men [aOR 2.31 95%CI 1.40-3.80], those with university studies [aOR 3.01 95%CI 1.04-8.69], temporary contracts [aOR 2.26 95%CI 1.29-3.98] and salaries between 751-1,200€ per month [aOR 1.74 95% CI 1.04-2.92] were more likely to report sickness presenteeism. Also, recent immigrants with good self-perceived health and good mental health were more likely to report presenteeism than Spanish-born workers with the same good health indicators.

**Conclusions:**

Immigrant workers report more sickness presenteeism than their Spanish-born counterparts. These results could be related to precarious work and employment conditions of immigrants. Immigrant workers should benefit from the same standards of social security, and of health and safety in the workplace that are enjoyed by Spanish workers.

## Background

Over the past two decades, important changes have taken place in the worldwide economy and consequently in labour markets [1,2]. One of these changes has been the conversion of full-time to temporary or flexible contracts and reductions in social protections for workers [1-4]. Additionally, the demographic profiles of many European countries have changed as they are receptors of immigrant workers primarily from low-income countries [5-7]. There are an estimated 30 million immigrants living in the European Union (EU-27), of which 19.5 million were citizens of countries outside of the EU27. Germany, Spain, United Kingdom, France and Italy are the countries with the greatest proportion of foreign-born population, according to Eurostat data (2008) [6]. In 2008, 17% of EU-27 immigrants were located in Spain [6]. The proportion of immigrants in Spain that come from low-income countries has increased from 0.7% to 8.3% between 1996 and 2008 [8]. Nevertheless, it is important to consider the fact that this figure may be biased downward, since those immigrants in an irregular situation (undocumented) are less likely to be included in standard registries and therefore may not appear in the official statistics data. In Spain, it is estimated that 2% of all immigrant workers could be in this situation of invisibility [3,9].

This new context in which immigration takes place makes it necessary to analyse the employment and working conditions of the immigrant population and the impact on health as a part of the research on determinants of health inequalities. Preceding studies have established that immigrant workers may face precarious work, employment difficulties, low salaries and lack of access to social benefits [10,11], together with increased risk for occupational health and safety problems [12-15].

In spite of indicators that show worse employment conditions, previous studies have observed that immigrants are less likely to miss work for a health problem when compared with the autochthonous population after adjustment for age and the duration of sick leave [16]. Among the explanations suggested by the literature include: a) the healthy migrant effect, by which immigrants arrive in host countries with relatively good physical and mental health status due to selection as part of the migratory process [17], and b) presenteeism, whereby workers go to work despite being sick [18]. However, relevant information concerning the determinants of presenteeism is still relatively sparse [19-22]. The aim of this study is to assess the extent of sickness presenteeism in a sample of Spanish-born and foreign-born workers according to personal and employment characteristics and taking into account time living in Spain as a proxy for the healthy migrant effect.

## Methods

### Design, data collection and setting

This cross-sectional study is a part of the larger ITSAL Project (*Inmigración, Trabajo y Salud*, for its Spanish acronym). A 74 item questionnaire was developed with the aim of gathering information on socio-demographic characteristics, migration processes, employment and working conditions, and physical and mental health of immigrants working in Spain (questionnaire available upon request) [15]. The questionnaire was developed based on the results of a previous qualitative phase of study in the ITSAL Project [13].

The sample of foreign-born workers (n = 2,434) consists of individuals from the countries providing the bulk of immigrants in Spain (Morocco, Ecuador, Romania and Colombia). The survey was carried out in four Spanish cities representing major places of immigrants' residence (Barcelona, Huelva, Madrid and Valencia) [8]. Inclusion criteria for the survey include having lived in Spain for at least one year; having worked in Spain for at least three months; not working as an athlete, artist, graduate student, or business executive; not being a Spanish citizen or married to a native Spaniard; and having Spanish language abilities sufficient to participate in the interview. Quota sampling was set by nationality, gender, and area of residence in Spain.

All participants were provided an informational letter explaining their rights and guaranteeing individual confidentiality. Participation was voluntary, with consent implied by the decision to complete the survey. Interviews with foreign-born workers (average length: 30 minutes) were carried out by professional interviewers, many of them from migrant sending countries, in locations frequently visited by immigrants (associations that ceded their space, cultural centres, meeting rooms in urban hotels, and in the participants' workplaces and homes) in the participating cities.

Within the larger group of immigrants, 1,841 individuals between 20 and 40 years old were included in the study. Interviews with immigrants were conducted with a 55.8% response rate.

The sample of Spanish-born workers (n = 509) was constructed to resemble the foreign-born sample according to gender, age (20-40 years old), and area of residence in Spain. Sampling took place in selected neighborhoods from the participant cities in which at least 15% of the population was foreign-born. Interviews were conducted with a 55.0% response rate.

The interviewers received specific training regarding the survey's objectives, as well as instruction in techniques for providing confidence to the interviewees.

The study protocol was approved by the Ethical Committees of the participating institutions (University Pompeu Fabra of Barcelona, University of Valencia, University of Huelva, University of Alicante and Trade Union Institute for Work, Environmental and Health of Madrid).

### Variable definitions

The outcome variable used was sickness presenteeism. It was measured through the responses to two questions: 1) Have you had health problems in the last year? and 2) Have you had to miss work for these health problems? Sickness presenteeism was determined to exist when a worker had a health problem being employed (yes, question 1) and had not missed work in spite of that problem (no, question 2). These questions refer to employment conditions in Spain (the most important job or the most recent job). The total number of workers who stated that they had had any health problem was 1,617 for foreign-born (78.5%) and 442 for Spanish-born (21.5%) workers. Analyses were conducted with this final sample.

Other variables such as sex, age (20-30, 31-40 years), education (without studies/primary, secondary and university), type of contract (permanent, temporary, no contract), average income (in Euros) in the last 3 months before the survey (<= 750, 751- 1200, 1201- 1800, >1800) were considered. Health indicators also were considered in the analysis: self-rated health and mental health. Self-rated health ("How would you rate your current health status?") was dichotomized as good (original responses: good or very good) or poor (original responses: fair, poor, or very poor). Mental health was measured with the 12-item version of the General Health Questionnaire GHQ-12 [23]. Responses were rated and summed, and individuals with a score of 3 or higher were classified with high probability of a psychiatric disorder (poor mental health) and the remaining (good). Both health measures have been extensively used and validated in previous studies [24,25].

### Analysis

Socio-demographic and occupational characteristics of foreign-born and Spanish born workers and the prevalence of sickness presenteeism were described for each category of personal and employment characteristics (and Chi square for proportions). In the case of foreign-born workers, analyses were stratified for length of stay in Spain (>= 2 years, < 2 years). This stratification served to control for the healthy migrant effect and the process of integration in the host country. Analyses for general trend in proportions considering the three categories (Spanish born; foreign born and >= 2 years; foreign born < 2 years) were carried out by each type of personal and employment variable by means of Chi square for trend using Epiinfo 3.5.1. Logistical regression models were used to compare presenteeism in both groups, first crudely and then adjusting by all the variables included in the study. Odds ratios with 95% confidence intervals were obtained. All calculations were computed using SPSS 17.0.

## Results

The prevalence of sickness presenteeism was higher in foreign-born workers than in Spanish-born workers, more noticeably in immigrants with a shorter time of residence in Spain (56.3%). The same patterns are shown when repeating analyses for each single category of sample subgroups according to the different variables, except in the subsample of interviewees with income equal or lower than 750 euros, in which presenteeism is higher in Spanish than in immigrant workers. Almost all the differences are significant (p < 0.05 for trend) except in the case of those with permanent or no contracts and those with an income equal to or lower than 750 euros (table 1).

**Table 1:** Sickness Presenteeism in foreign-born and Spanish born workers according to personal, employment and health characteristics (ITSAL Project, 2009; n = 2,059)

Variables	Spanish-born				Foreign-born								p-value for trend
					Time in Spain >=2 years				Time in Spain < 2 years				
	
	N^a^	n^b^	P^c^	p-value	N^a^	n^b^	P^c^	p-value	N^a^	n^b^	P^c^	p-value	
Sex													
Males	226	90	39.8	0.429	818	427	52.2	0.575	119	69	58.0	0.586	p < 0.0001
Females	216	95	44.0		586	297	50.7		94	51	54.3		p < 0.0001
Age (years)													
20-30	218	88	40.4	0.597	808	413	51.1	0.692	109	61	56.0	0.910	p < 0.0001
31- 40	224	97	43.3		596	311	52.2		104	59	56.7		0.008
Education^d^													
≤Primary	95	34	35.8	0.189	420	226	53.8	0.144	72	38	52.8	0.040	0.001
Secondary	208	85	40.9		736	362	49.2		110	58	52.7		0.020
University	139	66	47.5		246	136	55.3		31	24	77.4		0.002
Type of contract													
Permanent	209	84	44.4	0.090	382	178	46.6	p < 0.0001	58	29	50.0	0.325	0.110
Temporary	177	70	35.5		608	294	48.4		92	51	55.4		0.009
No contract	56	31	53.4		414	252	60.9		63	40	63.5		0.352
Income last 3 months^d^													
<= 750	68	41	60.3	0.004	417	236	56.6	0.024	64	40	62.5	0.142	0.318
751- 1200	180	72	40.0		737	376	51.0		112	60	53.6		0.005
1201- 1800	144	57	39.6		209	93	44.5		33	20	60.6		0.028
> 1800	46	13	28.3		39	17	43.6		3	0	0.0		0.052
Self-perceived health													
Good	385	169	43.9	0.024	1160	627	54.1	p < 0.0001	181	112	61.9	p < 0.0001	p < 0.0001
Poor	57	16	28.1		244	97	39.8		32	8	25.0		0.031
Mental health (GHQ-12)													
Good	324	150	46.3	0.002	1011	558	55.2	p < 0.0001	159	96	60.4	0.041	0.001
Poor	118	35	29.7		393	166	42.2		54	24	44.4		0.011
													
**Total**	**442**	**185**	**42.0**		**1404**	**724**	**51.6**		**213**	**120**	**56.3**		**p < 0.0001**

^a ^N = Number of workers reporting health problems in the last year.

^b ^n = Number of workers with health problems who not missed to work

^c ^P = Prevalence (n/N)

^d ^There was some missing information for education (n = 2, 0.09%) and income (n = 7, 0.33%)

After adjusting for the personal and employment variables included in the study, Foreign-born workers with less than 2 years of residence in Spain continue to report work presenteeism (aOR 1.77 95%CI 1.24-2.53) than their Spanish counterparts, especially in men (aOR 2.31 95%CI 1.40- 3.80), workers with university studies (aOR 3.01 95%CI 1.04-8.69), temporary contracted workers (aOR 2.26 95%CI 1.29- 3.98), and workers with salaries between 751 and 1,200 Euros per month (aOR 1.74 95%CI 1.04- 2.92) (table 2).

**Table 2:** Crude and adjusted OR Sickness Presenteeism in foreign-born workers according to personal, employment and health characteristics (ITSAL Project, 2009; n = 2,059).*

	Foreign-born workers							
	
Variables	Time in Spain >= 2 years				Time in Spain < 2 years			
	
	OR	IC95%	aOR^a^	IC95%	OR	IC95%	aOR^a^	IC95%
Sex								
Males	1.65	1.22- 2.23	1.52	1.11- 2.07	2.09	1.33- 3.27	2.31	1.40- 3.80
Females	1.31	0.96- 1.79	1.14	0.81- 1.61	1.51	0.93- 2.46	1.05	0.61- 1.83
Age (years)								
20-30	1.55	1.14- 2.09	1.40	1.12- 1.76	1.88	1.18- 2.99	2.18	1.26- 3.76
31- 40	1.43	1.05- 1.95	1.48	1.19- 1.84	1.72	1.07- 2.75	1.32	0.79- 2.23
Education								
<= Primary	2.09	1.32- 3.32	1.69	1.03- 2.78	2.01	1.07- 3.74	1.62	0.78- 3.36
Secondary	1.40	1.03- 1.91	1.38	1.00- 1.91	1.61	1.01- 2.57	1.49	0.91- 2.44
University	1.37	0.90- 2.08	1.25	0.80- 1.96	3.79	1.53- 9.38	3.01	1.04- 8.69
Type of contract								
Permanent	1.30	0.92- 1.83	1.27	0.89- 1.83	1.49	0.83- 2.67	1.57	0.84- 2.96
Temporary	1.43	1.02- 2.01	1.50	1.05- 2.12	1.90	1.14- 3.17	2.26	1.29- 3.98
No contract	1.25	0.72- 2.20	1.30	0.69- 2.45	1.40	0.67- 2.93	1.01	0.39- 2.57
Income last 3 months								
<= 750	0.86	0.51- 1.45	0.77	0.43- 1.36	1.10	0.54- 2.21	0.92	0.41- 2.04
751- 1200	1.56	1.12- 2.18	1.49	1.06- 2.08	1.73	1.08- 2.79	1.74	1.04- 2.92
1201- 1800	1.22	0.80- 1.88	1.18	0.74- 1.88	2.35	1.08- 5.09	2.19	0.91- 5.26
> 1800	1.96	0.80- 4.83	2.62	0.85- 8.10	N.A	N.A	N.A	N.A
Self-perceived health								
Good	1.50	1.19- 1.90	1.39	1.09- 1.77	2.08	1.45- 2.98	2.04	1.38- 3.00
Poor	1.69	0.90- 3.18	1.28	0.66- 2.51	1.17	0.44- 3.14	2.70	0.78- 9.38
Mental Health (GHQ-12)								
Good	1.43	1.11- 1.84	1.16	0.89- 1.53	1.77	1.20- 2.60	1.66	1.10- 2.51
Poor	1.73	1.11- 2.70	1.67	1.05- 2.92	1.90	0.97- 3.69	1.50	0.65- 3.41
								
Total	1.48	1.19- 1.84	1.40	1.24- 2.53	1.79	1.29- 2.49	1.77	1.24- 2.53

* In all categories, Spanish-born in the corresponding subgroup of workers is the reference group (1.00)

^a ^aOR: Adjusted OR by the rest of variables in the table

Among those workers reporting good self-perceived health, immigrants living in Spain less than 2 years were more likely to report presenteeism in comparison to their Spanish counterparts (aOR 2.04 95%CI 1.38- 3.00). Among workers reporting good mental health, immigrants (living less than 2 years in Spain) were more likely to report presenteeism than Spanish workers (aOR 1.66 95%CI 1.10- 2.51); in this same subcategory of workers, immigrants reporting poor mental health and living in Spain more than 2 years were more likely to report presenteeism than Spanish workers (aOR 1.67 95%CI 1.05- 2.92).

## Discussion

A higher prevalence of sickness presenteeism was found in foreign-born workers living in Spain less than 2 years, after adjusting for potential confounders, and these differences are relevant in all the different subcategories of workers considered according to sociodemographic, employment and health status. To the best of our knowledge, this is a first approach to the assessment of sickness presenteeism in immigrant workers.

The higher sickness presenteeism in immigrant workers in comparison with the Spanish population may be explained by greater exposure to precarious employment conditions among immigrants [11,15,26,27]. Interestingly, we observed more presenteeism in immigrants with temporary contracts than in immigrants with permanent contracts (in contrast to what was observed in Spanish workers). This situation highlights the relative importance of employment conditions (unfair or informal) in an already vulnerable population. This means that worse working and employment conditions are the pathway through which immigrants experience more health problems, especially after their second year in Spain. Therefore we would expect more sickness absence due to these health problems, but what we observe is more sickness presenteeism [28]. In a large proportion of cases, immigrant workers feel unprotected and vulnerable in the workplace, with less capacity to demand their rights [11,13]. In other cases, their lack of knowledge of the social protections and difficulty navigating the health system may also be a contributor [29]. In addition, it is important to consider other cultural factors that vary according to the countries included in the sample. For instance, different perceptions of risk and self-perception of health and illness may exist between native and foreign-born workers [30]. Furthermore, we found in previous qualitative research that self-perception of health among immigrants is linked to life circumstances that prioritize economic sustainability of the self and family unit [27].

Other studies carried out in Spain have shown that sickness absence is higher in autochthonous workers than in immigrants [16,31], and the same situation was found in our sample. A higher absenteeism was observed in Spanish workers in comparison with their foreign-born counterparts through additional analysis measured through the same questions from the survey.

The results of our study are not easily comparable with other research, due to the lack of studies of presenteeism in immigrants or that compare native-born and foreign-born workers. In the case of the few studies of presenteeism in the working population, the general prevalence found in our study- considering both immigrant and Spanish workers- is similar to that of one European study [22] in which 48% of men and 51% of women had to attend work at least once, despite being sick. In another study [19] higher rates of presenteeism were shown: 73% for women and 67% for men. In any case, because of the complexity of the topic and the indicators used in those studies, the comparison and the derived conclusions must be interpreted carefully, especially in terms of international comparisons. It is important to consider the key points suggested by the authors, for instance, the relationship between presenteeism and low monthly income and occupational social class and employment.

Despite these limitations, it is very relevant to study sickness presenteeism due to its social, economic and health implications [19,32,33], which include its association with loss of productivity, its association with poor organizational climate in the workplace and its possible relationship with future sickness absenteeism [20,32,34-36]. One of the advances made by this research is that we have evaluated presenteeism from a perspective of the social determinants of health, analysing how the inequalities faced by immigrants originate give rise to situations of vulnerability in host countries [15,37]. Presenteeism is an emerging concept that can be viewed as an inequalities index.

Interpretation of these results should take into account the study's limitations. The data for this study was collected under quota sampling methodology, and the potential effects on the ability to extrapolate findings to a larger population should be noted. The hidden nature of immigrant populations, especially the undocumented subgroup, has prevented a completely accurate description of the population of interest, prohibiting random probability sampling. We do not have information concerning non-response across the target groups. Response rates in other research in similar populations vary. For example, in the Angeles County Health Survey [38], the use of random digital dialing and computer-assisted telephone interviews in different languages obtained a response rate of 18%, and the Vietnamese American Community Issue Survey, a bilingual, computer-assisted phone interview of 500 Vietnamese American adults (18 and older) examining participation in political protests, obtained a response rate of 25.5% [39]. The slightly lower response rate of Spanish workers in this case could be due to their being targeted more often for surveys. The higher response rate among immigrants could be due to the fact that the immigrants might feel a sense of solidarity with the workers conducting the survey, who are immigrants themselves, and their desire to voice their opinions.

It should be mentioned that there is the possibility of misclassification in the variable presenteeism. The variable assumes that sick workers that did not miss work during the time period in question is due to presenteeism and not a possible lack of employment. In other cases, if health problems are mild, workers do not need to miss work. More research is needed to standardized items to measure sickness presenteeism. The cross-sectional design of the study avoids assessing causality in the observed relationships. Prospective studies following a cohort of foreign-born and Spanish-born workers are needed to overcome these limitations. It is possible that there are some unobservable individual characteristics that have an influence on both decision to migrate and the subsequent prevalence of sickness presenteeism. Nevertheless, considering the characteristics of the sample, we think that the interest variables would be distributed randomly in Spanish and foreign-born populations and this situation would confirm our main findings. Likewise, people with an acceptable level of Spanish language sufficient to answer the questions included in the questionnaire were selected, and for that reason this study also leaves out other immigrant groups. There are immigrant groups in Spain proceeding from sub-Saharan countries, Asia and Oceania, which were not included in this study due to technical difficulties and economic resource limitations. Also, it is important to include in successive analyses workers in other age groups than those included in this study.

The ITSAL survey was conducted at the end of an economic cycle characterized by high rates of employment. In fact, according to the Active Population Survey -EPA-, the employment activity rates of the immigrant population are higher the rates for the native-born in the four community regions and the cities covered by the survey [40]. For that reason, it will be interesting to see how the situation changes as economic dynamics impact a job market characterized by higher unemployment and lower job stability. As such, we suppose that the rate of presenteeism could be higher now than when the original study took place.

It is important to consider that the models do not control for other labor and working conditions in the cities where the study was carried out. For that reason it is important to conduct further research about the impact of specific labour market conditions on health conditions in Spanish and immigrant populations by means of a multilevel approach. Also, complementary analyses are currently being carried out by the research team focused of the impact of the economic crisis in European Countries and particularly in Spain. Our focus is on how economic changes have impacted the employment, working and health conditions in immigrants compared with the Spanish-born population.

## Conclusion

The higher presenteeism found in the immigrant population when compared with the Spanish-born population, and the demographic and working conditions that may be related to this finding, suggest inequalities that require discussion and debate in the field of public health. The implementation of specific policies to reduce social inequalities in health is clearly linked to developing strategies related to protecting the health of immigrant workers. Immigrant workers should be entitled to the same standards of social security, and of health and safety in the workplace, that Spanish workers enjoy. In addition, further study should explore ways to extend sickness leave and effective social protection to all workers in Spain as a potential strategy for preventing sickness presenteeism.

## Competing interests

The authors declare that they have no competing interests.

## Authors' contributions

All the authors participate in the ITSAL Project. All of them contribute with the data analysis, the written of the manuscript and the approbation of the final version to be submitted to the journal.

## Pre-publication history

The pre-publication history for this paper can be accessed here:

http://www.biomedcentral.com/1471-2458/10/791/prepub
